# Supplementary material for: A whole-animal phenotypic drug screen identifies suppressors of atherogenic lipoproteins
Source: eLife. 2026 Aug 27;14:RP105314. doi: 10.7554/eLife.105314 (PMC13521566; doi:10.7554/eLife.105314)
Supplement: Supplementary file 2 [file elife-105314-code1.zip › Output_data/dual_flashlight_labeledEnoxo.pdf]

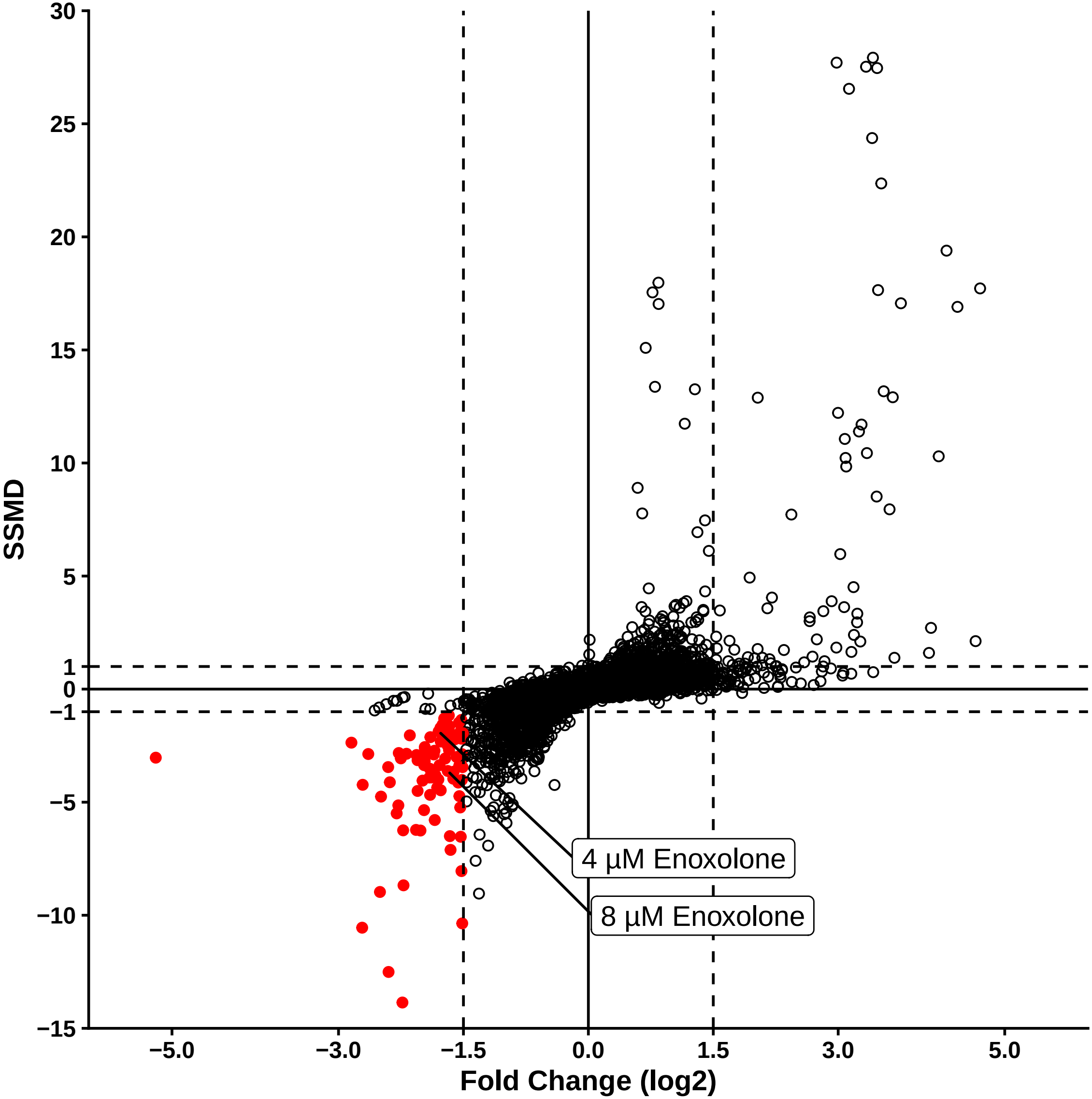

30
25
20
15
10
5
1
0
−1
−5
−10
−15
SSMD
−5.0
−3.0
−1.5
0.0
1.5
3.0
5.0
Fold Change (log2)
4 µM Enoxolone
8 µM Enoxolone
